# Nitrate enhances the secondary growth of storage roots in *Panax ginseng*

**DOI:** 10.1016/j.jgr.2022.05.009

**Published:** 2022-06-02

**Authors:** Kyoung Rok Geem, Jaewook Kim, Wonsil Bae, Moo-Geun Jee, Jin Yu, Inbae Jang, Dong-Yun Lee, Chang Pyo Hong, Donghwan Shim, Hojin Ryu

**Affiliations:** a Department of Biology, Chungbuk National University, Cheongju, Republic of Korea; b Department of Biological Sciences, Chungnam National University, Daejeon, Republic of Korea; c Ginseng & Medicinal Plant Research Institute, Chungnam Agricultural Research & Extention Service, Keumsan, Republic of Korea; d Department of Herbal Crop Research, National Institute of Horticultural and Herbal Science, Rural Development Administration, Eumseong, Republic of Korea; e Korea Ginseng Corporation, R&D Headquarters, Daejeon, Republic of Korea; f Theragen Bio Co., Ltd, Suwon, Republic of Korea; g Department of Biological Sciences and Biotechnology, Chungbuk National University, Cheongju, Republic of Korea

**Keywords:** Nitrate, Root secondary growth, Hormone, Panax ginseng

## Abstract

**Background:**

Nitrogen (N) is an essential macronutrient for plant growth and development. To support agricultural production and enhance crop yield, two major N sources, nitrate and ammonium, are applied as fertilizers to the soil. Although many studies have been conducted on N uptake and signal transduction, the molecular genetic mechanisms of N-mediated physiological roles, such as the secondary growth of storage roots, remain largely unknown.

**Methods:**

One-year-old *P. ginseng* seedlings treated with KNO_3_ were analyzed for the secondary growth of storage roots. The histological paraffin sections were subjected to bright and polarized light microscopic analysis. Genome-wide RNA-seq and network analysis were carried out to dissect the molecular mechanism of nitrate-mediated promotion of ginseng storage root thickening.

**Results:**

Here, we report the positive effects of nitrate on storage root secondary growth in *Panax ginseng*. Exogenous nitrate supply to ginseng seedlings significantly increased the root secondary growth. Histological analysis indicated that the enhancement of root secondary growth could be attributed to the increase in cambium stem cell activity and the subsequent differentiation of cambium-derived storage parenchymal cells. RNA-seq and gene set enrichment analysis (GSEA) revealed that the formation of a transcriptional network comprising auxin, brassinosteroid (BR)-, ethylene-, and jasmonic acid (JA)-related genes mainly contributed to the secondary growth of ginseng storage roots. In addition, increased proliferation of cambium stem cells by a N-rich source inhibited the accumulation of starch granules in storage parenchymal cells.

**Conclusion:**

Thus, through the integration of bioinformatic and histological tissue analyses, we demonstrate that nitrate assimilation and signaling pathways are integrated into key biological processes that promote the secondary growth of *P. ginseng* storage roots.

## Graphical abstract

**Figure:**
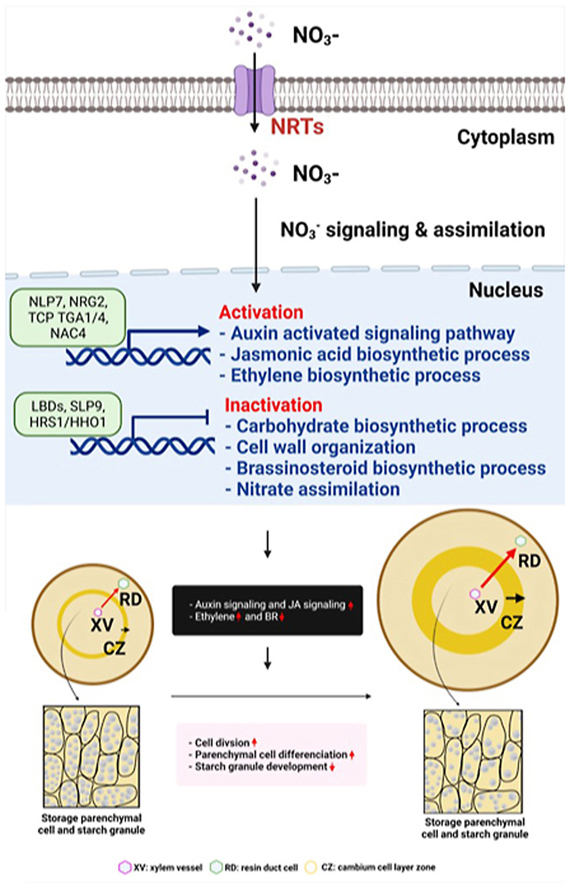

## 1 Introduction

*Panax ginseng* Meyer, also called Korean ginseng, has long been used as an important herbal medicine to treat various diseases in Asian countries, especially Korea, China, and Japan [1,2]. Recent studies show that *P. ginseng* plants contain various types of pharmacologically active components called ginsenosides that exhibit many therapeutic effects because of their anti-allergic, antidiabetic, anticancer, anti-aging, and immunity- and vitality-enhancing properties [3,4]. Although *P. ginseng* has been used as an important medicinal root crop for thousands of years, the genetic and physiological factors affecting the growth and development of its storage roots have not been well characterized [1,5]. Therefore, many studies have been conducted recently to shed light on the growth and developmental characteristics of *P. ginseng*.

Being sessile organisms, terrestrial plants need to change their physiology to adapt to a variety of adverse environmental conditions. Nutrients are one of the key fundamental factors affecting the plant response to environmental fluctuations for modulating growth and development. Various inorganic elements influence a variety of plant physiological processes, thus affecting their growth and development. Nitrogen (N) is an essential macronutrient for plants. The natural availability of N is one of the main factors limiting plant productivity [6,7]. Most plant species require approximately 20–50 g N per kg dry weight to promote root growth [6]. The uptake and assimilation of N enhance the productivity of various root crops such as radish, carrot, cassava, and ginseng, and promote the accumulation of useful compounds required for plant growth and development [8]. N is present in the soil in the ionic form, i.e., nitrate (NO_3_^−^) and ammonia (NH_4_^+^), and as nitrogenous organic compounds [9]. Ammonium mainly stimulates lateral root initiation, whereas nitrate promotes lateral root elongation. In addition, nitrate treatment enhances shoot primary growth, root secondary growth, and nitrate assimilation related pathways. After its uptake and assimilation, nitrate is particularly used for the synthesis of amino acids and nucleic acids by nitrate transporters (NRTs) and nitrate reductases (NRs), respectively [[11], [12], [13]]. N availability enhances the photosynthetic efficiency of plants and the storage of carbon compounds by modulating the synthesis of organic acids, starch, and sucrose, which are required for plant growth and development [14,15]. However, the physiological and genetic roles of N assimilation in the growth and development of *P. ginseng* plants are largely unknown.

During the domestication of root crops, plants were mainly selected for their ability to accumulate useful compounds and energy sources in storage roots. Secondary root growth is also important for the productivity of root crops. *P. ginseng* is a slow-growing plant with a 4–6 year cultivation period; therefore, root secondary growth is particularly important for its productivity [16]. The control of root secondary growth is closely related to cambium stem cell maintenance, which is mainly controlled by the integrated signaling networks of plant hormones such as auxin and cytokinin [17,18]. Previous studies showed that various plant hormones and their crosstalk affect the division and differentiation of cambium stem cells. In a recent study, gibberellin (GA) treatment increased the division of xylem and fiber cells in *P. ginseng* by promoting cambium activity [16]. Genome-wide transcriptome analysis showed that GA enhances root secondary growth in *P. ginseng* by promoting cell division and cell wall biogenesis. Interestingly, genes involved in nitrate assimilation are tightly associated with the GA signaling network in GA-treated *P. ginseng* roots (16). Furthermore, N is closely related to plant hormone homeostasis and the signaling outputs of cytokinin (CK) and auxin [[19], [20], [21], [22]]. Exogenous nitrate treatment directly stimulates the auxin receptor AFB3, which activates auxin signaling pathways by promoting the degradation of Aux/IAA transcriptional repressors in the presence of auxin [22, 23]. CK signaling pathways are tightly connected with *NRT* gene expression and nitrate distribution in plants. These findings suggest that the physiological response of plants to N, the main limiting factor affecting plant growth and development, is closely linked to phytohormone-mediated signaling pathways.

Many factors involved in root secondary growth and closely related to the agricultural productivity of root crops have been investigated to date [17,19,[24], [25]]. In this study, the physiological effects of N on the secondary growth of storage roots of ginseng were evaluated. Externally applied nitrate significantly enhanced the primary and secondary growth of *P. ginseng* shoots and roots. The integrated microscopic histology and bioinformatic analysis of KNO_3_-treated ginseng roots revealed that the formation of a transcriptional network comprising genes controlling growth-promoting and stress-related hormones such as auxin, brassinosteroid (BR), jasmonic acid (JA), and ethylene is closely connected with the secondary growth of storage roots. In particular, the signaling networks of these hormones were associated with the downregulation of cell wall organization and carbohydrate biosynthesis. Interactions among these phytohormone signaling networks inhibited the accumulation of starch granules in storage parenchymal cells. Overall, these results provide new insights into the relationship between different forms of N and the mechanism controlling secondary growth of storage roots in *P. ginseng*.

## 2 Materials and methods

### 2.1 Plant materials

**Fig. 1:**
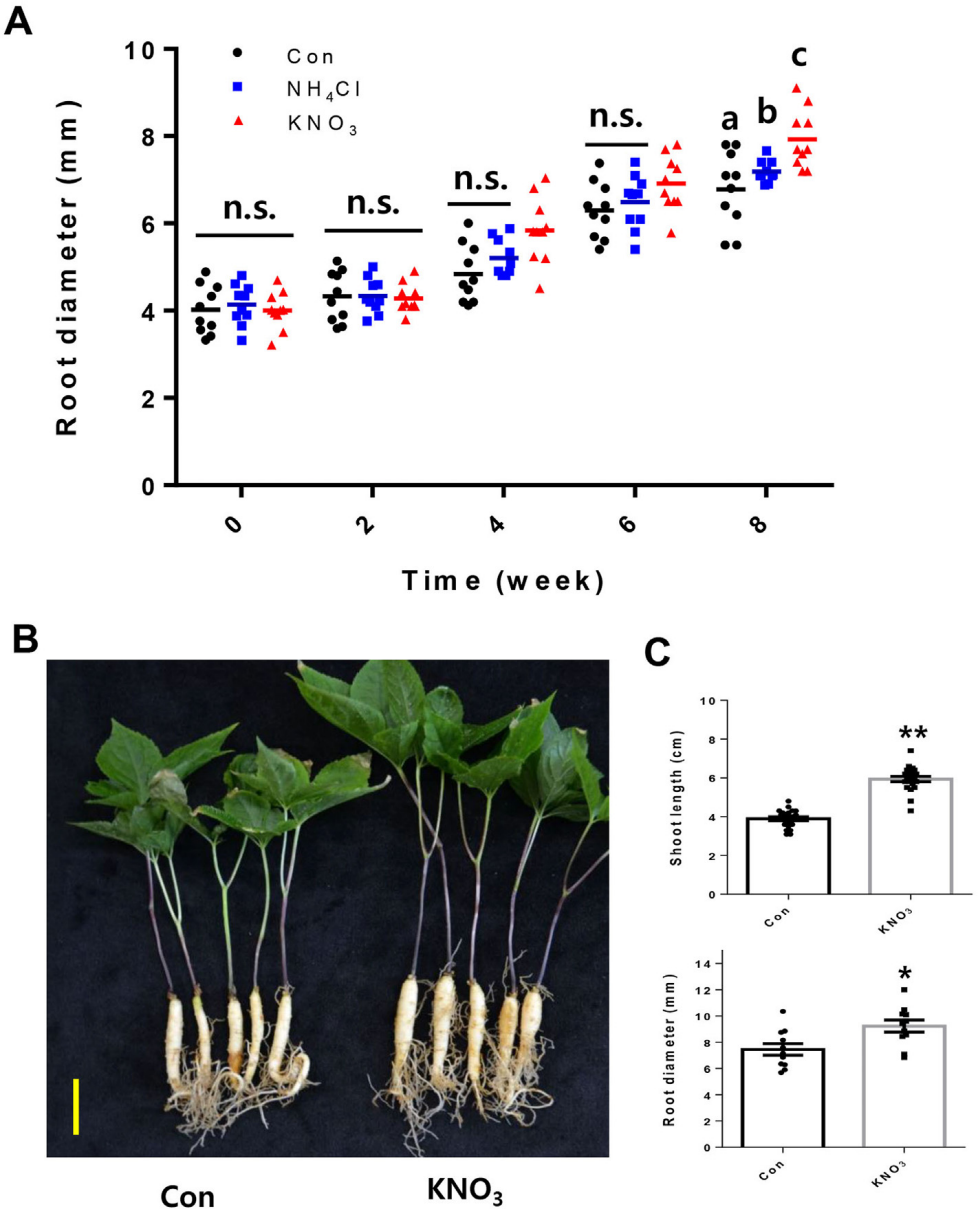
**Potassium nitrate (KNO**_**3**_**) treatment promotes shoot primary growth and root secondary growth in *P. ginseng*.** (A) Measurement of the diameter of one-year-old *P. ginseng* roots treated with a mock control (Con), 5 mM ammonium chloride (NH_4_Cl) or potassium nitrate (KNO_3_) once a week for 8 weeks. Error bars represent standard error (n = 10). Different lowercase letters indicate statistically significant differences *P* < 0.05; one-way analysis of variance [ANOVA], followed by Tukey's multiple range test). (B) Phenotype of 1-year-old *P. ginseng* plants treated with a mock control (Con) or 5 mM potassium nitrate (KNO_3_) once a week for 8 weeks. Scale bar = 2 cm. (C) Measurement of shoot length and root diameter of Fig. 1B. Dots in graphs represent individual values. Error bars represent standard error; *n* = 10 (∗*P* < 0.05, ∗∗P > 0.01, The significance of the difference was analyzed by *t*-test method.).

One-year-old *P. ginseng* seedlings (Yunpoong, provided by National Institute of Horticultural and Herbal Science) were grown at 22–23 °C with a 16 h light/8 h dark cycle into the ginseng cultivation soil medium in green house. Two weeks after transplantation of the one-year-old ginseng seedlings on dedicated soil (pH:5.21, EC: 0.07 ds/m, NO_3_–N: 5.54 mg/l, P_2_O_5_: 72.38 mg/l, Cham-Grow Inc.) for *P. ginseng* cultivation, a control (Mock/water), ammonium chloride (NH_4_Cl) or potassium nitrate (KNO_3_) was treated once a week for 8 weeks with a soaking method. The *P. ginseng* roots were soaked until completely submerged for 10 min. To determine optimal N source concentration for *P. ginseng* root growth, we initially conducted a series of treatments at 1, 2.5, 5, and 10 mM of KNO_3_ and NH_4_Cl. The gradual increments of storage root growth of *P. ginseng* were only observed in KNO_3_ up to 5 mM, but not in 10 mM condition and NH_4_Cl treatment (Fig. 1A and data not shown). We finally treated 5 mM of KNO_3_ and 1 mM of NH_4_Cl. After a total of eight treatments, the ginseng storage tap roots were analyzed for the developmental patterns of secondary growth.

### 2.2 Histological sections and microscopy

To get the histological section images of *P. ginseng* root and shoot samples, we carried out the paraffin sectioning. This paraffin sectioning method followed as described in our previous work (Hong et al., 2021). The fresh samples of *P. ginseng* roots and shoots were fixed in 1% glutaraldehyde and 4% formaldehyde in PBS pH 7.0 at 4 °C overnight. The tissues were dehydrated in 30%, 50%, 70%, 90% and 100% EtOH three times, 1 h each and then the samples were embedded in paraffin. The 5-10 μm-thick sections of samples with a microtome were mounted onto slides and stained with 1% Safranin-O (SIGMA, cat. S2255) and 0.5% Astra blue (Santa-cruz biochem., cat. sc-214558 A). Bright and polarized light images from *P. ginseng* samples using a Slideview scanner and a BX53 microscope were obtained (Olympus). The root cell number was counted on a straight line derived by cambium layers from resin ducts to the inner xylem vessel cells.

### 2.3 RNA-seq analysis

Total RNA was extracted from the main storage root samples (a 2 cm sample taken from the top region of the tap roots of *P. ginseng*) that were treated with mock and KNO_3_ four times over the course of four weeks using an Easy Spin RNA Extraction Kit (iNtRON Biotechnology) with the manufacturer's instructions. Total RNA was prepared for RNA-seq libraries with three biological replicates, using TruSeq Stranded mRNA Library Prep Kit (Illumina, Inc., San Diego, CA). cDNA was synthesized and subjected to end repair, single ‘A’ addition and adapter ligation. The libraries were purified and enriched via PCR amplification, and then sequenced on the Illumina HiSeq 4000 platform to generate 100-bp paired-end reads (Supplementary Table S1). The Raw data were deposited in the NCBI Short Read Archive database under the accession number; PRJNA792481.

### 2.4 Differentially expressed genes analysis

Paired-end reads were cleaned using prinseq-lite version (0.20.4) with the following parameters: min_len 50; min_qual_score 5; min_qual_mean 20; derep 14; trim_qual_left 20; trim_qual_right 20 [26]. Clean paired-end reads of each sample were aligned to the ginseng reference sequences using Bowtie 2 [27]. The RSEM 1.3.0 software was used to obtain read counts and TMM-normalized TPM (trimmed mean of M value-normalized transcripts per million) values for each transcript [28]. EdgeR version 3.16.5 was used to calculate the negative binomial dispersion across conditions for differential gene expression analysis [29]. Genes were determined to be significantly differentially expressed if they showed a false discovery rate (FDR)-adjusted P < 0.05 [30]. To validate the RNA-seq results, cDNA was synthesized using the TOP script™ RT Dry MIX (Enzynomics, Cat. no. RT200). Quantitative real-time reverse transcription-polymerase chain reaction (qRT-PCR) was performed using the KOD SYBR qRT MIX (TOYOBO) to validate the transcripts level of Gene Set Enrichment Analysis (GSEA) transcriptome data which is down- or up-regulated genes by KNO_3_ treatment. *PgACT* was used as an internal control for qRT-PCR (Fig. S3). All used primers are listed in Supplementary Table S6. Ginseng reference sequences were obtained from Ginseng Genome Database (http://ginsengdb.snu.ac.kr/index.php).

### 2.5 Functional annotation and network analysis

BLAST program with e-value threshold of 1 E^−5^ against *Arabidopsis thaliana* protein database was used for functional annotation of differentially expressed genes. Gene Ontology (GO) term enrichment analysis was performed using DAVID and enriched GO term was determined by Fisher Exact test (P < 0.05) [31,32]. Enriched GO genes were further analyzed with GSEA, as described in our previous work [33]. Network analysis was performed with GeneMANIA app in Cytoscape [34,35]. Bar graph was visualized with ggplot2 package in project R [36]. In the enrichment plot, the red line represents the gene subset that made the largest contribution to the enrichment score (ES). The ranking list metric in the plot measures the correlation between a gene and the plant phenotype. In the ranking list, positive values indicate genes up-regulated in mock-treated control root samples with red color gradient, and negative values indicate genes down-regulated in the mock-treated root samples.

## 3 Results

### 3.1 Exogenous nitrate application promotes shoot growth and root secondary growth in *P. ginseng*

N is an essential, yet limited, macronutrient for plants, and accounts for approximately 60% of the total fertilizer applied to crops each year. Plants absorb and utilize N in two major forms: nitrate and ammonium. To understand the physiological effects of nitrate and ammonium on *P. ginseng* growth, fully germinated 1-year-old *P. ginseng* seedlings were treated with 5 mM of KNO_3_ or NH_4_Cl once a week for 8 weeks (Fig. 1A). During the first 2 weeks, the secondary growth of *P. ginseng* roots was similarly increased in both N treatments. However, after four weeks of treatment, the rate of increase in root diameter was considerably greater in the KNO_3_ treatment than in the mock or NH_4_Cl treatments (Fig. 1A). These results suggest nitrate exerts a stronger effect on promoting the secondary growth of *P. ginseng* storage roots than ammonium. Next, we investigated the positive effect of nitrate-mediated growth in *P. ginseng* in further detail. The root diameter and shoot length of KNO_3_-treated *P. ginseng* seedlings were significantly enhanced by approximately 23% and 65%, respectively, compared with those of mock-treated (control) seedlings (Fig. 1B).

**Fig. 2:**
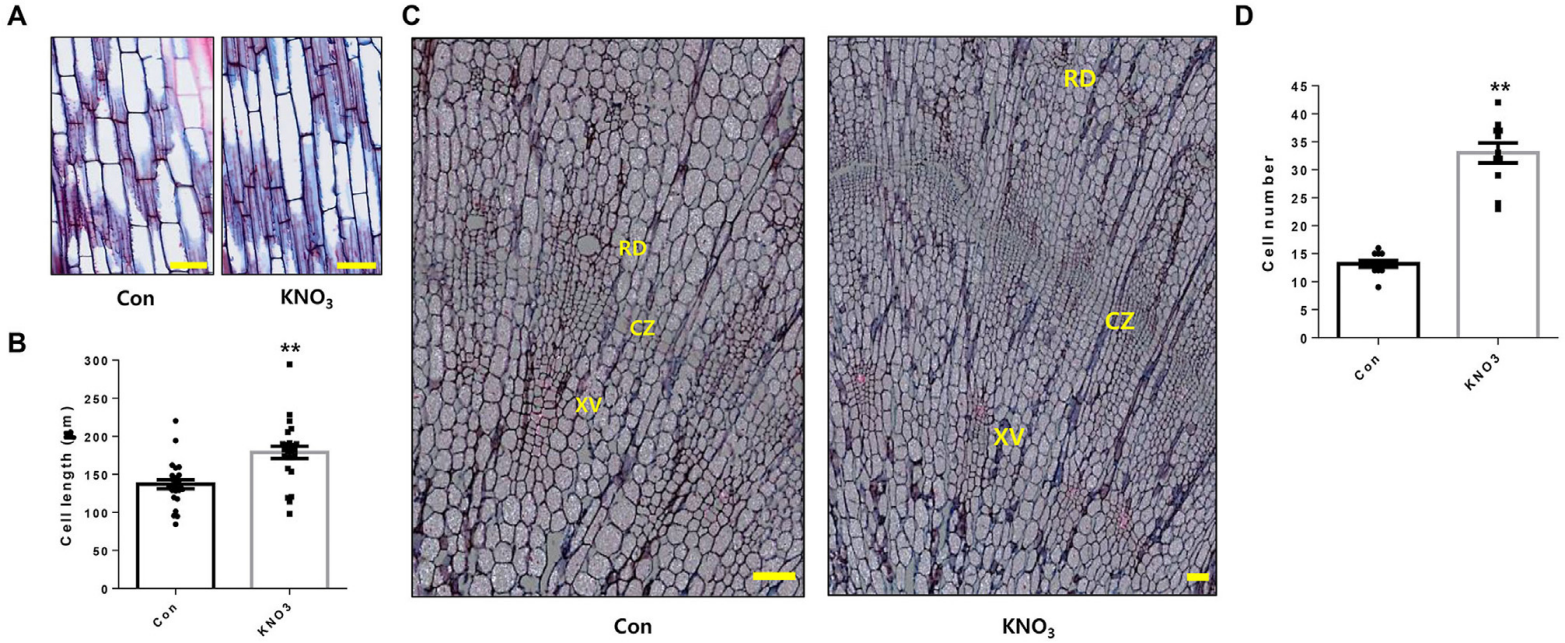
**Histological sections of KNO**_**3**_**-treated shoots and roots of *P. ginseng*.** (A) Representative stem images of stained stem cross-sections of *P. ginseng* plants treated with DMSO (Con) or 5 mM KNO_3_. Scale bar = 100 μm. (B) Quantification cell length in (A) was measured using ImageJ software. (C) Representative root images of stained stem cross-sections of *P. ginseng* plants treated with DMSO (Con) or 5 mM KNO_3_. XV: Xylem vessel, CZ: Cambial cell layer zone, RD: Resin duct cells. Scale bar = 100 μm. (D) Quantification of cambium-derived cells in XV and RD of each ray. Dots in graphs represent individual values. Error bars represent standard error; *n* = 25 (B), 11 (D). (∗*P* < 0.05, ∗∗P > 0.01, The significance of the difference was analyzed by *t*-test method.).

To gain additional insight into the physiological effects of KNO_3_ on *P. ginseng* growth, we conducted a histological analysis of paraffin-embedded sections of ginseng shoots and roots by staining with safranine-Astra blue combination. We found that KNO_3_-treated *P. ginseng* shoots had approximately 30% longer epidermal cells than mock-treated shoots (Fig. 2A and B). Moreover, the KNO_3_ treatment greatly improved the development of storage parenchyma cells from cambium stem cells located in the cambial zone [CZ] of the storage tap root (Fig. 2C). Consistently, the number of divided starch-deposited storage parenchyma cells positioned between the xylem vessels (XV) and resin duct cells (RD) was significantly increased by more than 2-fold in KNO_3_-treated *P. ginseng* roots compared to controls. (Fig. 2C and D).

### 3.2 Nitrate enhances cambial stem cell activity in ginseng roots

**Fig. 3:**
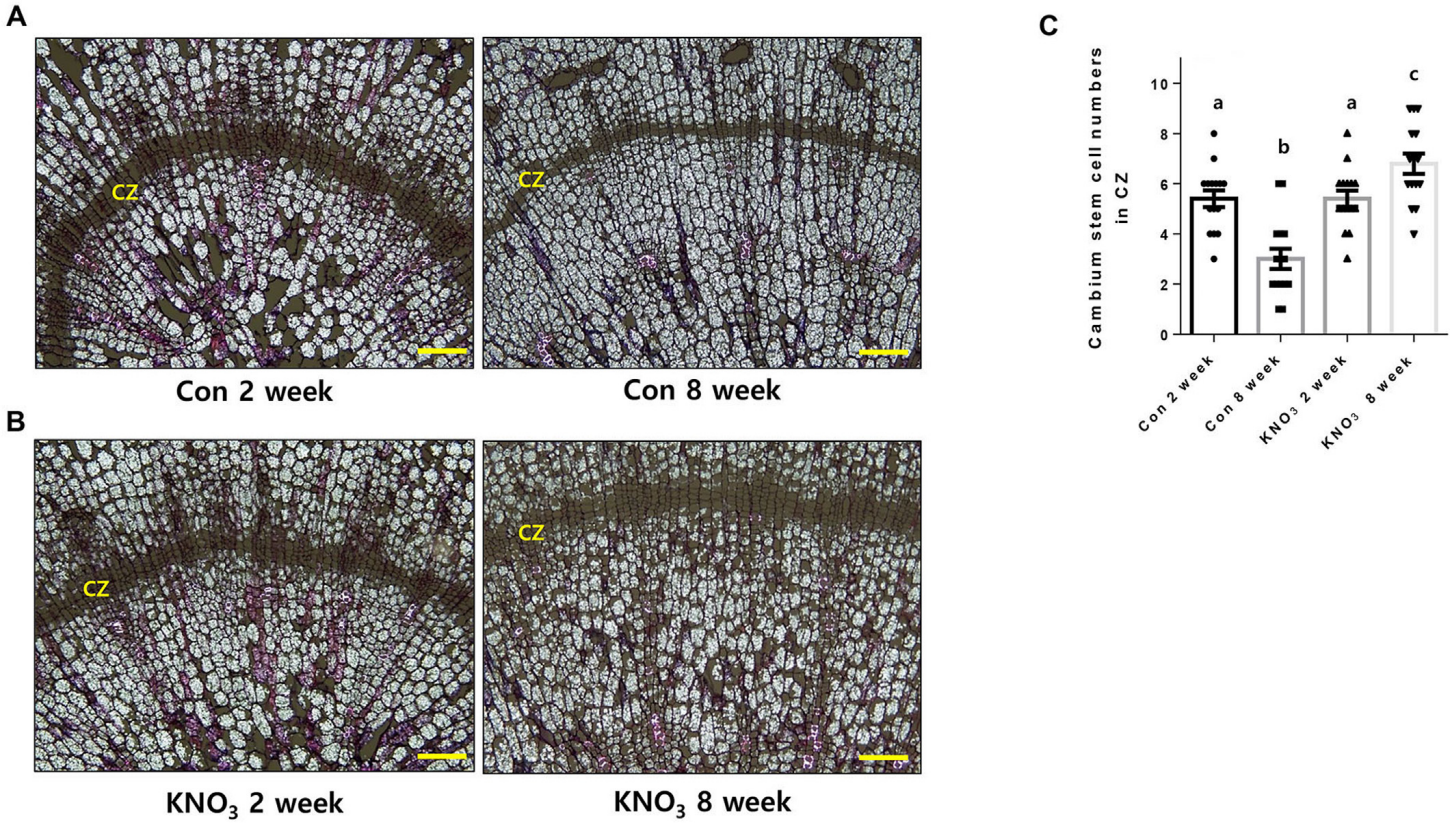
KNO_3_ treatment enhances the cambial stem cell activity of ginseng roots. (A, B) Phenotype of the cambial stem cells of 1-year-old *P. ginseng* roots treated with control (A) and 5 mM KNO_3_ (B) for two and eight weeks. Scale bar = 100 μm. (C) The numbers of cambial stem cells in the cambial cell layer zone (CZ). Error bars represent standard error (n = 15). Different lowercase letters indicate statistically significant differences *P* < 0.05; one-way ANOVA, followed by Tukey's multiple range test.

The radial secondary growth of storage roots is mainly governed by cambium stem cell activity. Here, we investigated the effect of exogenously applied nitrate on cambial stem cell maintenance. The dividing meristematic stem cell numbers in the CZ were significantly reduced in mock control after 8 weeks compared with 2-week-old control roots (Fig. 3A and C). However, treatment of ginseng plants with KNO_3_ for 8 weeks significantly enhanced their cambial cell activity in roots (Fig. 3B and C). The maintenance of cambial cell activity increased the number of cambium-derived storage parenchymal and vascular cells by enhancing cell division (Fig. 3B). These results indicate that nitrate-treated *P. ginseng* roots exhibit higher cambium stem cell activity compared with the mock control. Together, these results suggest that nitrate facilitates root secondary growth by enhancing the maintenance of cambial stem cell activity and subsequently the differentiation of storage parenchyma cells.

### 3.3 Transcriptome analysis of *P. ginseng* storage roots

**Fig. 4:**
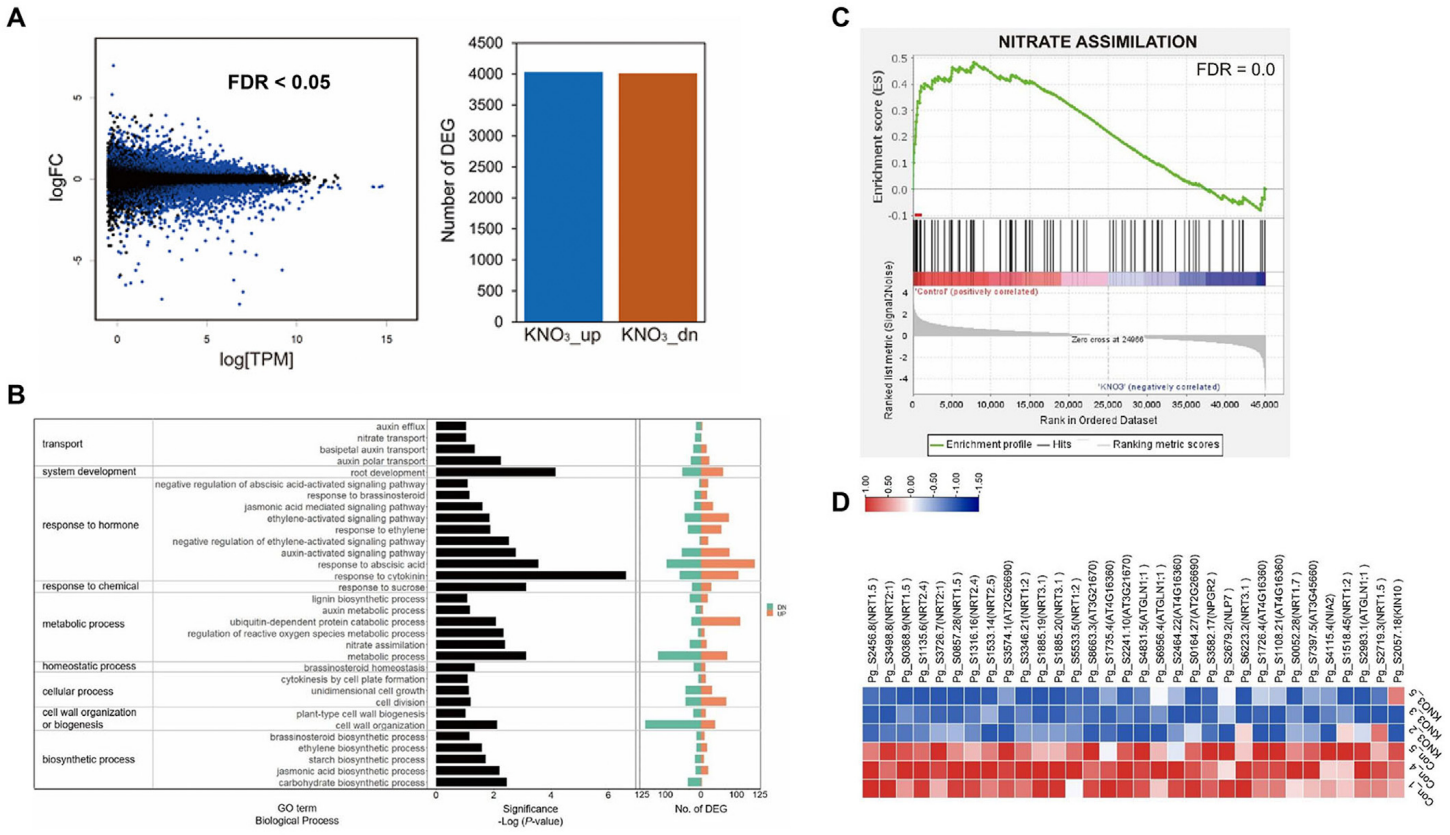
**Transcriptome profiling of *P. ginseng* roots grown with or without KNO**_**3**_**.** (A) MA plot of differential expression between mock-and KNO_3_-treated samples. Blue dots and bar graph represent the either up-(4030) and down-(4011) regulated genes with *q* < 0.05 and ≥ |1.5|-fold change. (B) Gene ontology (GO) enrichment analysis of differentially expressed genes (DEGs) identified by comparison of control and KNO_3_-treated root samples. GO terms in biological process of level 3 and level 5, with EASE score <0.01, were selected (left panel). The number of up-regulated genes (red) and down-regulated genes (green) categorized under the enriched GO terms are shown in the right panel. (C) Enrichment plot for a nitrate assimilation (GO:0042128) in the transcriptome data of control and KNO_3_-treated ginseng root samples. In the enrichment plot, the red dotted line (leading-edge subset) represents the gene subset that made the largest contribution to the enrichment score (ES) (false discovery rate [FDR] < 0.05). The ranking list metric in the plot measures the correlation between a gene and the plant phenotype. In the ranking list, positive values indicate genes up-regulated in mock-treated root samples with red color gradient, and negative values indicate genes down-regulated in mock-treated root samples. (D) Expression heatmap of the leading-edge subset genes (red line) contained in the nitrate assimilation related GO term.

To investigate the mechanism of nitrate-induced secondary growth in *P. ginseng* storage roots, we carried out RNA-seq analysis of ginseng roots sampled from the mock and KNO_3_ treatments. A total of 8041 differentially expressed genes (DEGs) were identified, of which 4030 were upregulated and 4011 were downregulated (Fig. 4A and Table S1). Gene ontology (GO) enrichment analysis of the DEGs revealed that nitrate treatment was significantly associated with the following functional categories: ‘transport’ (*p*-values of sub-GO terms in the representative category: *p* = 0.035 for auxin efflux; *p* = 0.035 for nitrate transport), ‘response to hormone and chemical’ (*p* = 0.00098 for auxin-activated signaling pathway; *p* = 0.012 for JA-mediated signaling pathway), ‘metabolic process’ (*p* = 0.0015 for nitrate assimilation), ‘cellular process’ (*p* = 0.048 for unidimensional growth; *p* = 0.046 for cell division), ‘biosynthetic process’ (*p* = 0.009 for ethylene biosynthetic process; *p* = 0.0013 for carbohydrate biosynthetic process), and ‘cell wall organization or biogenesis’ (*p* = 0.0052 for cell wall organization) (Fig. 4B). Additionally, gene set enrichment analysis (GSEA) revealed that a nitrate assimilation-related term (false discovery rate [FDR] = 0.0) was negatively enriched in the KNO_3_-treated ginseng roots (Fig. 4C). We identified 32 critical leading-edge subset genes, which were enriched gene set group leading to enrichment scores (ES) with respect to expression changes (Fig. 4D and Table S2). These leading-edge subset genes, including *NRT*s and N-responsive genes (*NLP*s, *GLN*s, and *NIA*s), were significantly downregulated in KNO_3_-treated samples (Fig. 4D). These results suggest that nitrate assimilation-related genes are regulated by a negative feedback pathway in the presence of sufficient nitrate.

### 3.4 Identification of key hormonal pathways involved in nitrate-mediated root secondary growth

**Fig. 5:**
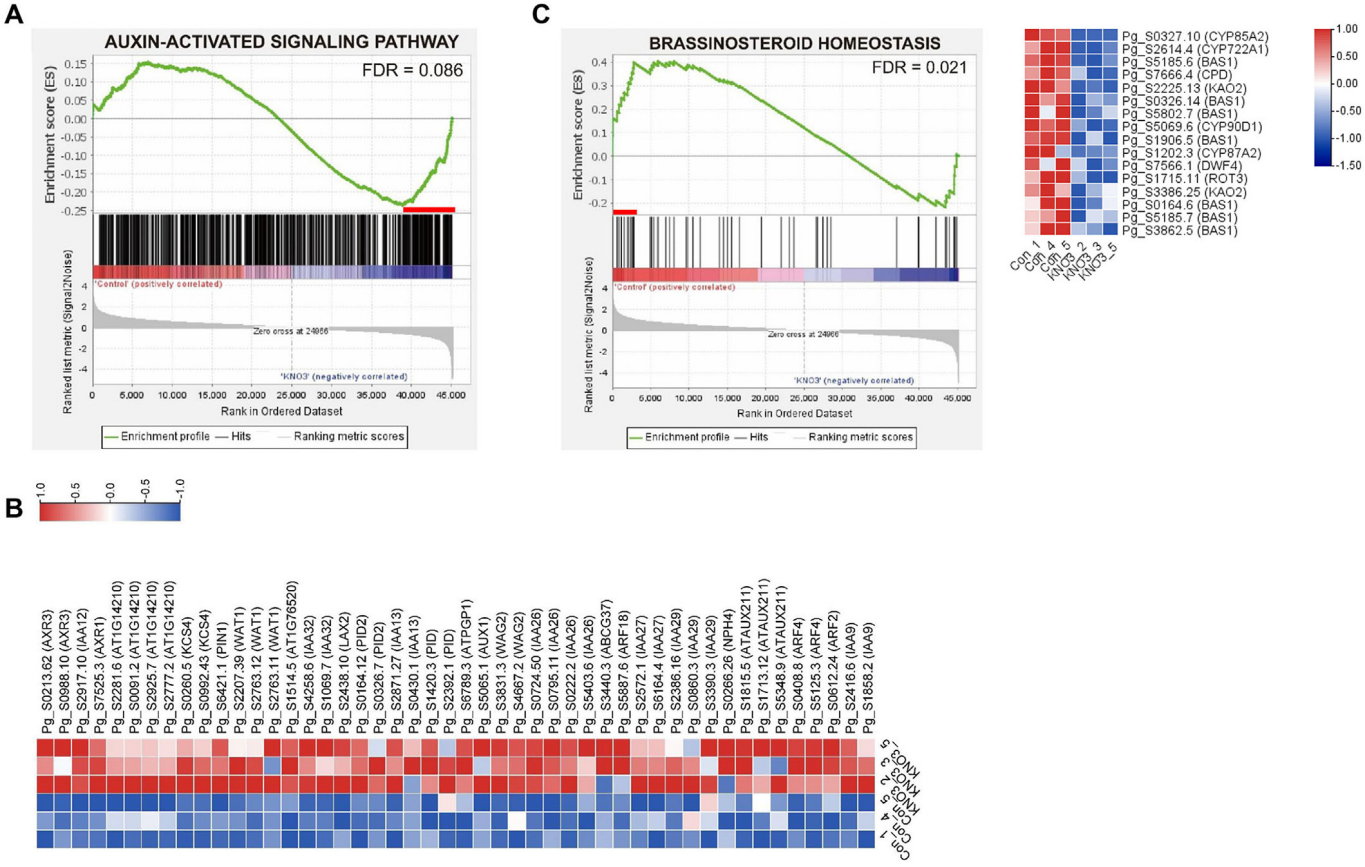
**Functional enrichment of auxin-activated signaling pathway and brassinosteroid homeostasis related terms in the nitrate-mediated secondary growth promotion of *P. ginseng* root.** (A, B) Enrichment plot for the auxin-activated signaling pathway (A, GO:0009734, FDR = 0.086), and an expression heatmap of leading-edge subset genes related to this pathway (B). (C) Enrichment plot for the brassinosteroid homeostasis (GO:0010268, FDR = 0.021), and an expression heatmap of leading-edge subset genes. Red lines indicate the leading-edge subset genes in the GSEA.

GO enrichment analysis indicated the involvement of auxin transport-related and hormone-responsive genes such as CK, JA and ethylene in the nitrate-mediated promotion of ginseng root secondary growth (Fig. 4). Moreover, the cambium activity was strongly maintained in the KNO_3_-treated samples (Fig. 3). Recent studies show that Walls Are Thin 1 (WAT1)-mediated local auxin accumulation and subsequent signaling activation in xylem precursor cells are critical events for the induction of plant secondary growth [37]. According to the results of GSEA, an auxin-activated signaling pathway term was significantly enriched among genes involved in auxin transport (*WAT1s, AUX1s, ABCG*, and *PID*s) and signaling (*ARF*s and *IAA*s) (Fig. 5A and B). Upregulation of several *WAT1* genes encoding a tonoplast-localized auxin efflux carrier in the xylem precursor cells by nitrate treatment (Fig. 5B) suggests that local auxin signaling activation in cambium stem cells facilitates the secondary growth of *P. ginseng* roots. It has been reported that auxin signaling has an important effect on the transport and biosynthesis of BRs [38]. Consistently, a BR homeostasis-related term (FDR = 0.021) was significantly enriched among the DEGs (Fig. 5C). Suppression of BR biosynthesis-related genes in the leading-edge subset by nitrate treatment indicates that upstream auxin signaling activation reduces the BR content of *P. ginseng* roots (Fig. 5C and Table S2). The negative correlation between BR homeostasis and root thickness is further supported by our previous finding, where exogenous BR application reduced root secondary growth in *P. ginseng* [39]. The CK related DEGs were not significantly enriched in the GSEA of a nitrate-mediated ginseng root thickening process (FDR = 0.667, Fig. S1A, Table S3). Also, CK signaling genes such as histidine kinases, response regulators, and cytokinin response factors were down-regulated in the KNO_3_-treated samples (Fig. S1B). However, genes involved in cambium development and homeostasis, such as *SVP, PTL*, and *WAT1*, were upregulated in nitrate-treated ginseng roots, correlating with the increased cambium activity (Fig. 3 and Fig. S2A) [40,41]. These findings, along with co-expression patterns of some epidermal-specific genes (Fig. S2B), suggest that nitrate assimilation is critical in the maintenance of cambium activity and secondary growth of *P. ginseng* roots via auxin and BR signaling pathways.

**Fig. 6:**
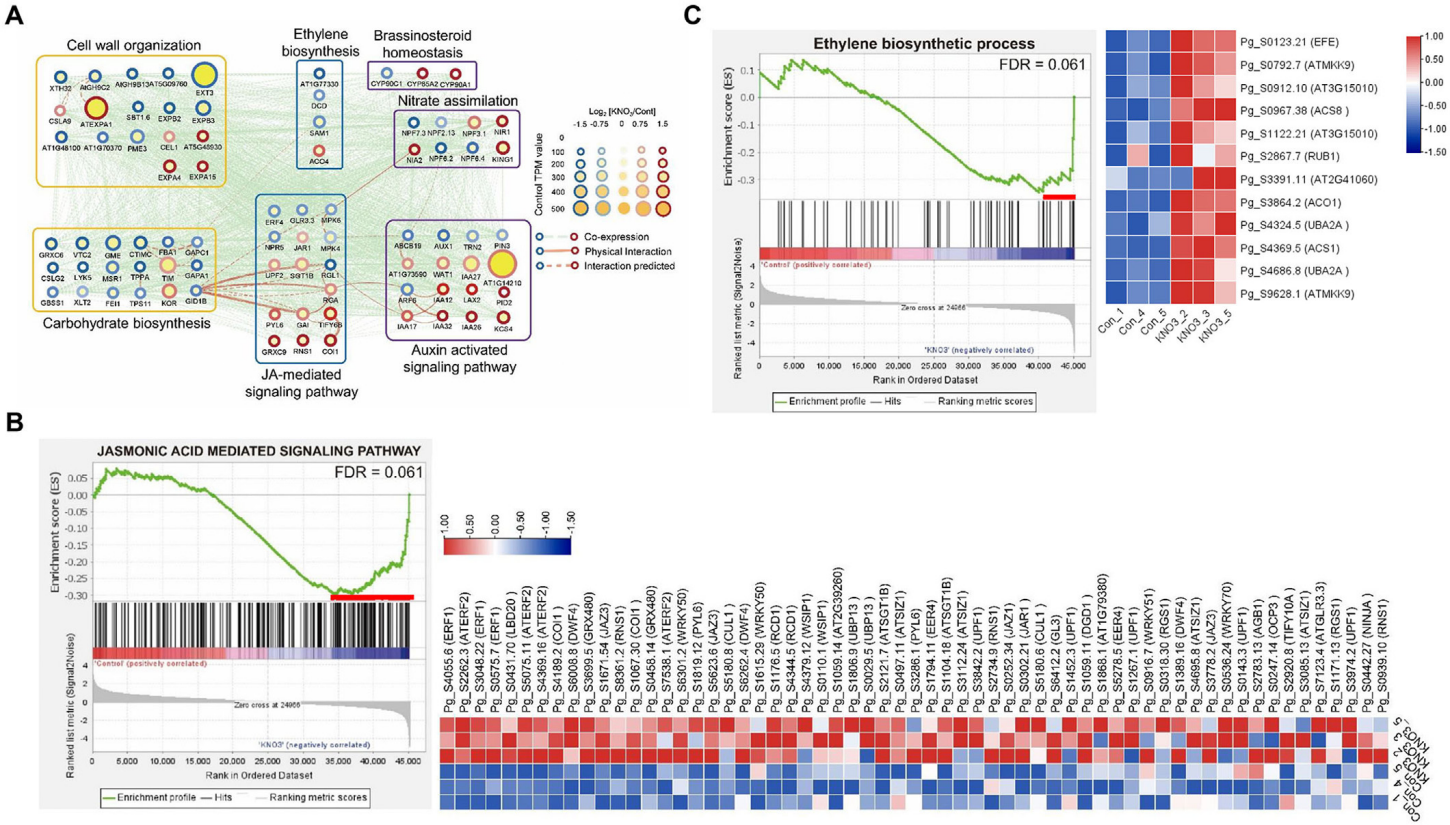
**Protein-protein interaction (PPI) network analysis of DEGs reveals other important pathways for nitrate-mediated secondary growth of ginseng roots.** (A) PPI network analysis of DEGs. (B, C) Enrichment plot for the jasmonic acid mediated signaling pathways (B, GO:0009867, FDR = 0.061) and ethylene biosynthetic process (C, GO:0009693, FDR = 0.061), and expression heatmaps of leading-edge subset genes. Red lines indicate the leading-edge subset genes in the GSEA.

Next, we focused on the identification of the key signaling network associated with auxin- and BR-related gene sets during root secondary growth. The selected auxin-activated pathway and BR homeostasis-related genes were subjected to transcriptional network analysis, based on the homology to *Arabidopsis thaliana* genes and its protein–protein interaction network using the STRING database (Fig. 6A). Network analysis revealed that the auxin-activated signaling pathway was tightly connected with ethylene, JA, cell wall organization, and carbohydrate biosynthesis (Fig. 6A and Table S4). GSEA also showed that the activation of genes related to ethylene biosynthesis and JA-mediated signaling (FDR = 0.061) were critical members of the leading-edge subset in nitrate-treated ginseng roots (Fig. 6B, C and Table S2). Consistent with this result, JA and ethylene have a positive effect on promoting plant secondary growth by stimulating cambium stem cell division [42]. These results suggest that the nitrate-mediated promotion of root thickness in *P. ginseng* is likely achieved through the control of auxin, BR, JA, and ethylene signaling and interaction among these hormones. Taken together, these results suggest that nitrate positively controls root secondary growth through a transcriptional network comprising genes involved in auxin, BR, ethylene, and JA signaling crosstalk and homeostasis.

### 3.5 Nitrate negatively regulates carbohydrate biosynthesis and storage parenchymal cell division

**Fig. 7:**
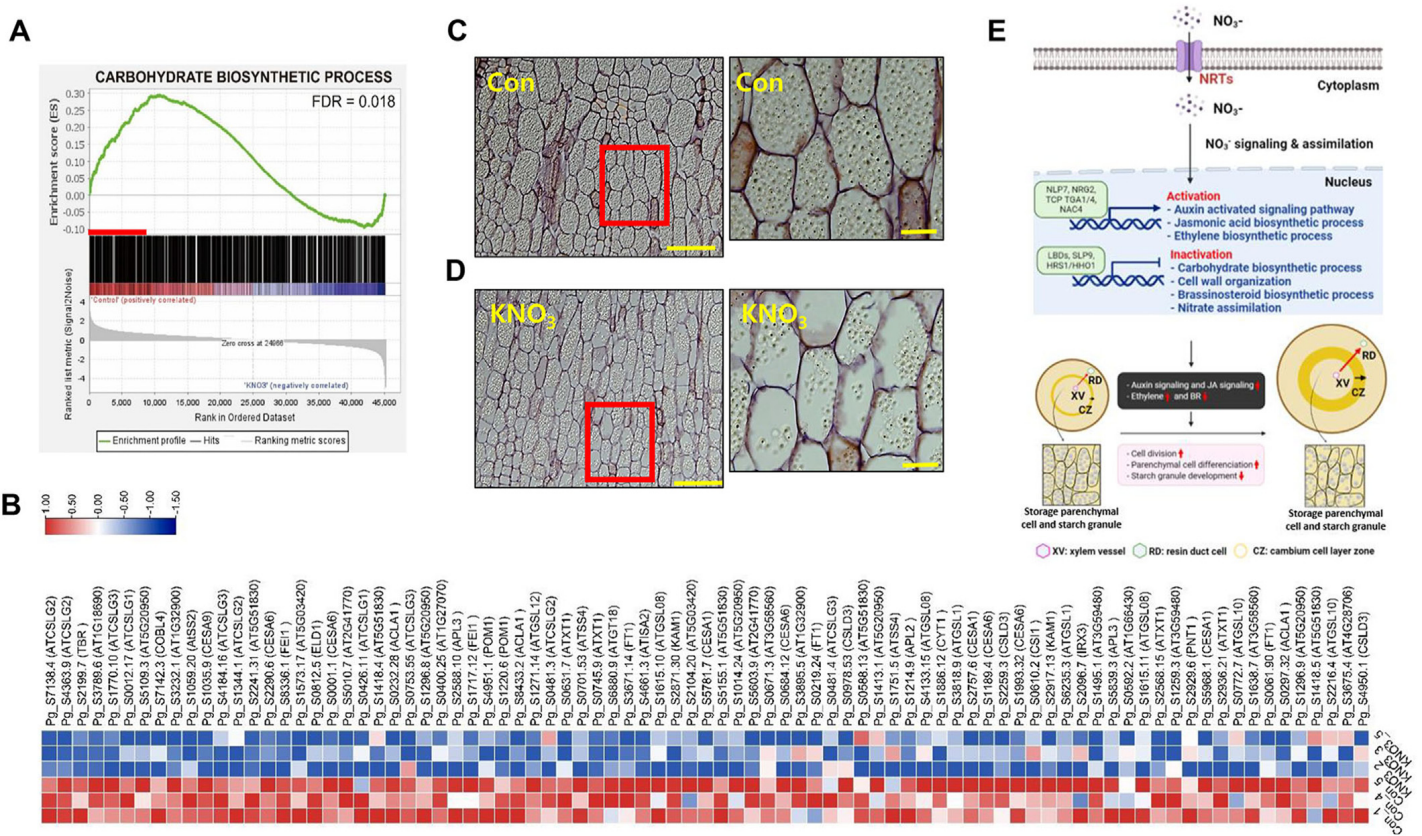
**Nitrate negatively regulates carbohydrate biosynthetic process and storage parenchymal cell development in ginseng roots.** (A, B) Enrichment plot for the carbohydrate biosynthetic process (A, GO:0016051, FDR = 0.086), and an expression heatmap of leading-edge subset genes related to this pathway (B). (C, D) Starch granule development in storage parenchyma cells of *P. ginseng* tap roots grown with (C) or without (D) 5 mM KNO_3_. (E) A schematic model for nitrate-mediated transcriptional network for promoting secondary growth of *P. ginseng* storage root (The model was created with BioRender.com).

Analysis of the transcriptional network further revealed that cell wall organization and carbohydrate biosynthesis exhibit a negative correlation with the nitrate-mediated increase in root thickness (Fig. 6A). We performed additional GSEA to identify major genes involved in this negative correlation. Fig. 7A shows that the carbohydrate biosynthetic process term was negatively correlated with nitrate treatment. Furthermore, many carbohydrate and cell wall metabolism-related genes, including *CESAs*, *CSLGs*, *GLSs*, and *CSLDs*, were greatly downregulated by nitrate treatment (Fig. 7B). Finally, we validated the expression pattern of selected DEGs by real-time qRT-PCR (Fig. S3). These results led us to hypothesize that nitrate treatment negatively affects the development of starch granules in the storage parenchymal cells. To test this hypothesis, we investigated the degree of starch granule development in KNO_3_-treated *P. ginseng* roots. The results showed that the KNO_3_ treatment promoted the differentiation of parenchymal cells in ginseng roots but inhibited the development of starch granules in storage parenchymal cells (Fig. 7C and D). Consistently, GSEA revealed that starch biosynthesis terms were mostly negatively correlated with KNO_3_-treated ginseng samples (ES = 0.225 in mock treatment), but starch degradation-related terms showed an opposite correlation (ES = −0.241 in KNO_3_ treatment; Figures S4A, S4B and Table S5). RNA-seq data on the expression levels of starch synthesis and degradation-related genes were further confirmed by qRT-PCR and linear regression analysis (R = 0.86; Fig. S4C-S4F). Taken together, our results indicate that nitrate facilitates the secondary growth of *P. ginseng* roots but negatively regulates the accumulation of starch granules.

## 4 Discussion

Extensive research has been performed over the past few decades on N as a macronutrient essential for plant growth and development [17]. As a major component of fertilizers used in agriculture fields worldwide, N is consumed for approximately 60% each year. Scientific advances have improved our understanding of N biogeochemical cycling, transport into plant cells, assimilation, diverse signaling crosstalk, and integration of plant growth and development [13]. Under terrestrial aerobic conditions, nitrate is the predominant N source in the soil and is mainly absorbed by NRTs present in plant root cells. Studies on N fertilizers have mainly been focused on cultivation methods that increase crop yield. In addition, studies have been conducted on the utilization of N sources through the efficient assimilation of the absorbed nitrate [43]. Many studies related to nitrate signaling at the molecular level have led to significant advances in recent years and have contributed greatly to the development of strategies for the efficient utilization of available nitrate. However, little is known about how nitrate assimilation and signaling pathways are integrated into the plant growth and developmental signaling network. In this study, we elucidated the physiological roles of nitrate signaling and assimilation in promoting the secondary growth of *P. ginseng* storage roots as well as the formation of a transcriptional network involved in this process (Fig. 7E). Our results revealed that nitrate is mainly involved in promoting primary and secondary growth in *P. ginseng* (Fig. 1, Fig. 2). In addition, the maintenance of cambium meristematic stem cell activity by nitrate treatment is critical for enhancing parenchyma cell differentiation in storage roots (Fig. 3). These physiological effects were further supported by evidence from RNA-seq analysis showing that the growth-promoting hormones, auxin and BR, interact with JA and ethylene responses (Fig. 4, Fig. 5, Fig. 6).

The complex hormonal regulation of cambium stem cell activity during the secondary growth of plants has been extensively investigated to date [[17], [18], [19],^25^,^43^]. Auxin and CK play essential roles in promoting secondary growth by facilitating xylem differentiation and cambium stem cell proliferation, respectively [17,18]. Our RNA-seq and GSEA results showed that the promotion of radial growth of roots in nitrate-treated ginseng plants was closely related to the activation of auxin signaling output (Fig. 4, Fig. 5). Nitrate uptake and assimilation promote polar auxin transport and root development [[44], [45],^46^], suggesting that nitrate treatment directly modulates auxin signaling activity. Interestingly, nitrate enhanced the expression of genes involved in the signaling and biosynthesis of JA and ethylene, respectively, two secondary growth-promoting plant hormones (Fig. 6). Transcriptional network analysis revealed that the interaction among these hormones is closely related to nitrate assimilation. A recent study reported that nitric oxide (NO) produced through the denitrification process under N fertilizer supplement has a positive effect on the colonization of roots by plant growth-promoting rhizobacteria (PGPR) and on improving the nitrogen use efficiency of crop plants [47]. Ethylene and JA play essential roles in root colonization of the commensal PGPR as well as cambium cell maintenance [42, 47]. More detailed studies are needed to elucidate how JA and ethylene, well-known stress hormones, facilitate the radial growth of ginseng storage roots, together with other growth-promoting hormones or PGPR, in a nitrate-rich environment.

Interestingly, our GO enrichment analysis showed that the GO term ‘response to cytokinin’ was most significantly enriched in the biological process category (Fig. 3B). Although CK signaling and biosynthesis-related genes were not significantly selected in the GSEA (Fig. S1A), many CK-related genes were differentially regulated in nitrate-treated ginseng roots. Many previous studies reported the essential roles of CK in the maintenance of cambium stem cells and sink activity. Interestingly, the expression patterns of cytokinin output related genes (response regulators, cytokinin receptors, CRFs etc) were reduced in nitrated treated ginseng roots (Figure S1B). Consistently, the increased meristematic activity of cambial cells facilitated storage parenchyma cell proliferation, but not starch granule development, in the nitrate-treated ginseng roots (Fig. 2, Fig. 6). These results suppose that the nitrate-mediated root thickening is not likely correlated with the CK-mediated root sink activity. Additionally, the antagonistic interactions of CK responses with auxin and GA during plant growth and development have been well established [17,18]. Previously, we reported the positive role of GA in the secondary growth of *P. ginseng* storage through the activation of secondary cell wall development- and organization-mediated transcriptional network [16]. Although the present study showed similar root radial growth-promoting effects of nitrate and GA, the suppression of cell wall organization, carbohydrate and starch biosynthesis in nitrate-treated ginseng roots delayed starch granule deposition in the growing storage parenchymal cells (Fig. 7 and Fig. S4). This difference in storage parenchymal cell development between nitrate- and GA-treated ginseng roots is probably caused by the opposing activity of CK in these different conditions. The relationship between nitrate signaling and CK-mediated responses during storage root secondary growth should be critically investigated at the molecular level to improve the knowledge of N fertilizers and their application in the field.

## Author contributions

D.S and H.R designed the experiments and supervised this study. K.R.G and W.B carried out plant growth experiments and histological sectioning analysis. K.R.G, W.B, M.G.J, J.Y, I.J., and D.Y.L prepared one-year-old ginseng seedlings and nitrate treatment analysis. J.K and D.S and C.P.H analyzed RNA-Seq data and bioinformatic analysis. K.R.G, J.K, C.P.H, D.S and H.R wrote the manuscript.

## Declaration of competing interest

The authors declare no conflict of interest.
