# Supplementary material for: Nitrate enhances the secondary growth of storage roots in Panax ginseng
Source: J Ginseng Res. 2022 Jun 2;47(3):469–78. doi: 10.1016/j.jgr.2022.05.009 (PMC10214138; doi:10.1016/j.jgr.2022.05.009)
Supplement: Multimedia component 2 [file mmc2.pdf]

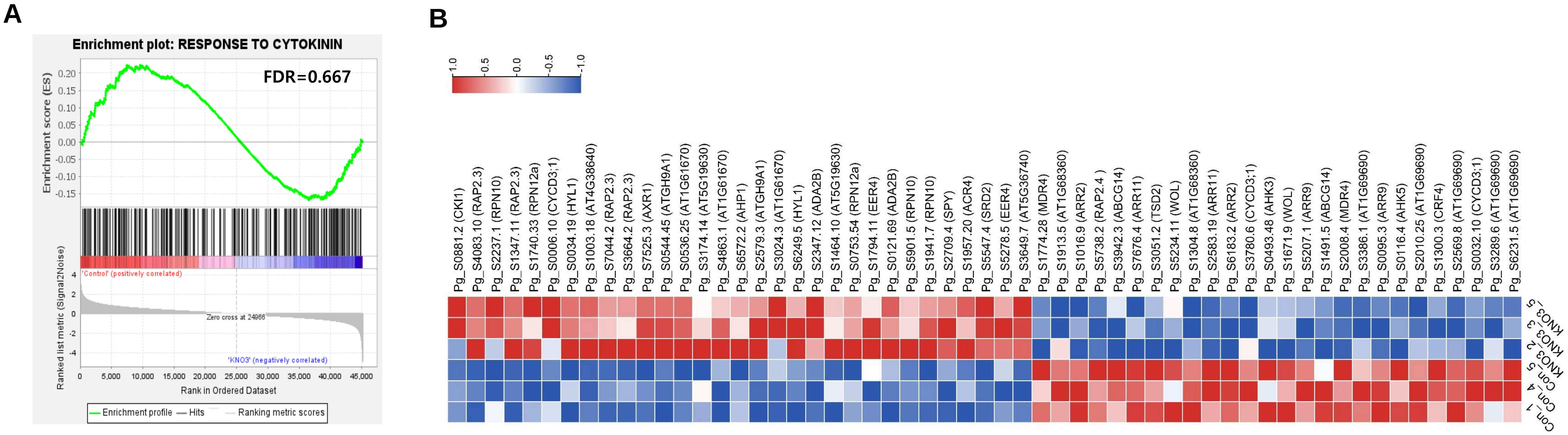

Supplementary Figure S1. KNO_3 treatment induces transcript level changes in subset of cytokinin responsive genes. (A) GSEA analysis with GO term “response to cytokinin” (GO:0009735). (B) Heatmap of DEGs which are included in GO term “response to cytokinin”.

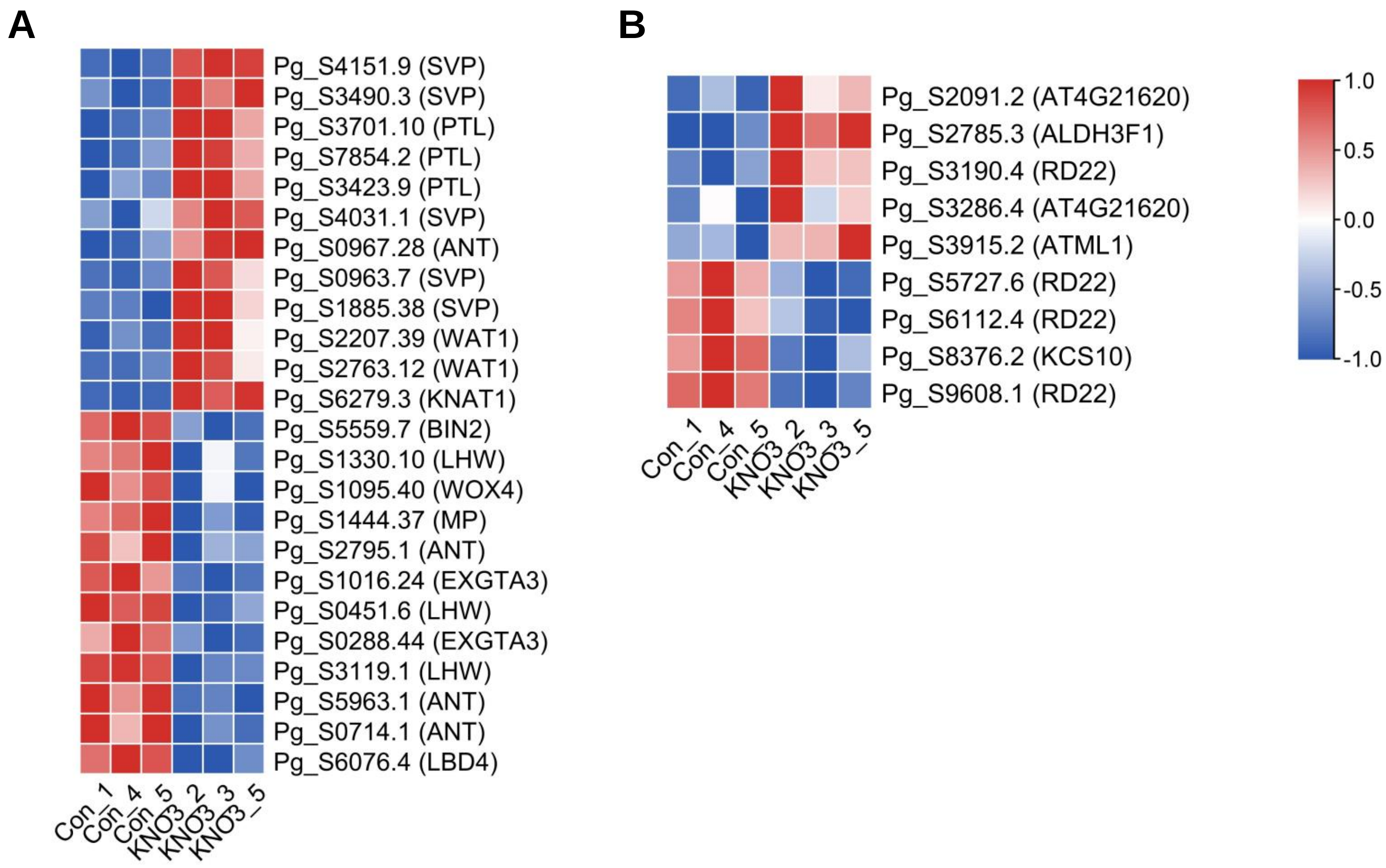

Supplementary Figure S2. KNO_3 treatment induces transcript level changes in subset of genes involved in cambium development or epidermis tissue. (A) Heatmap of DEGs which are included in cambium development related genes. (B) Heatmap of DEGs which are known to specific expressed genes in epidermal tissue.

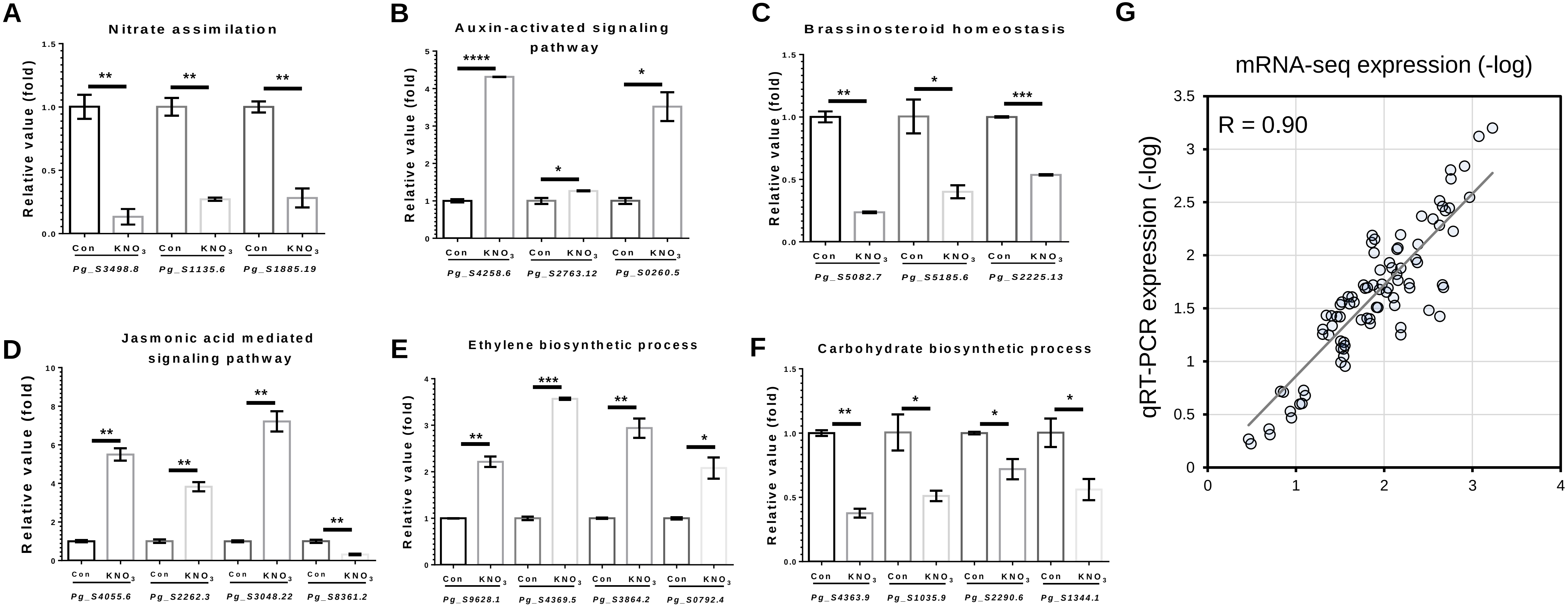

Supplementary Figure S3. Validation of the expression level of GSEA transcriptome data by qRT-PCR. Total RNAs were prepared from the root of *p. ginseng* with or without treatment of KNO_3. (A) The expression level of nitrate assimilation, (B) auxin-activated signaling, (C) Brassinosteroid homeostasis, (D) jasmonic acid-mediated signaling pathway (E) ethylene biosynthetic process, and (F) carbohydrate biosynthetic process relate genes were analyzed by quantitative real-time reverse transcription-polymerase chain reaction (qRT-PCR). *PgACT* was used as an internal control. Error bars indicate SEM (*P < 0.05, **P < 0.01 ***P < 0.001, ****P < 0.0001 Student's t-test).(G) Linear regression analysis between qRT-PCR expression and mRNA-seq data. Relative expression levels of each gene were determined by 2−ΔΔCT method, as indicated in the x axis, while the TMM-normalized TPM of target genes divided by that of *PgACT* is indicated in the y axis.

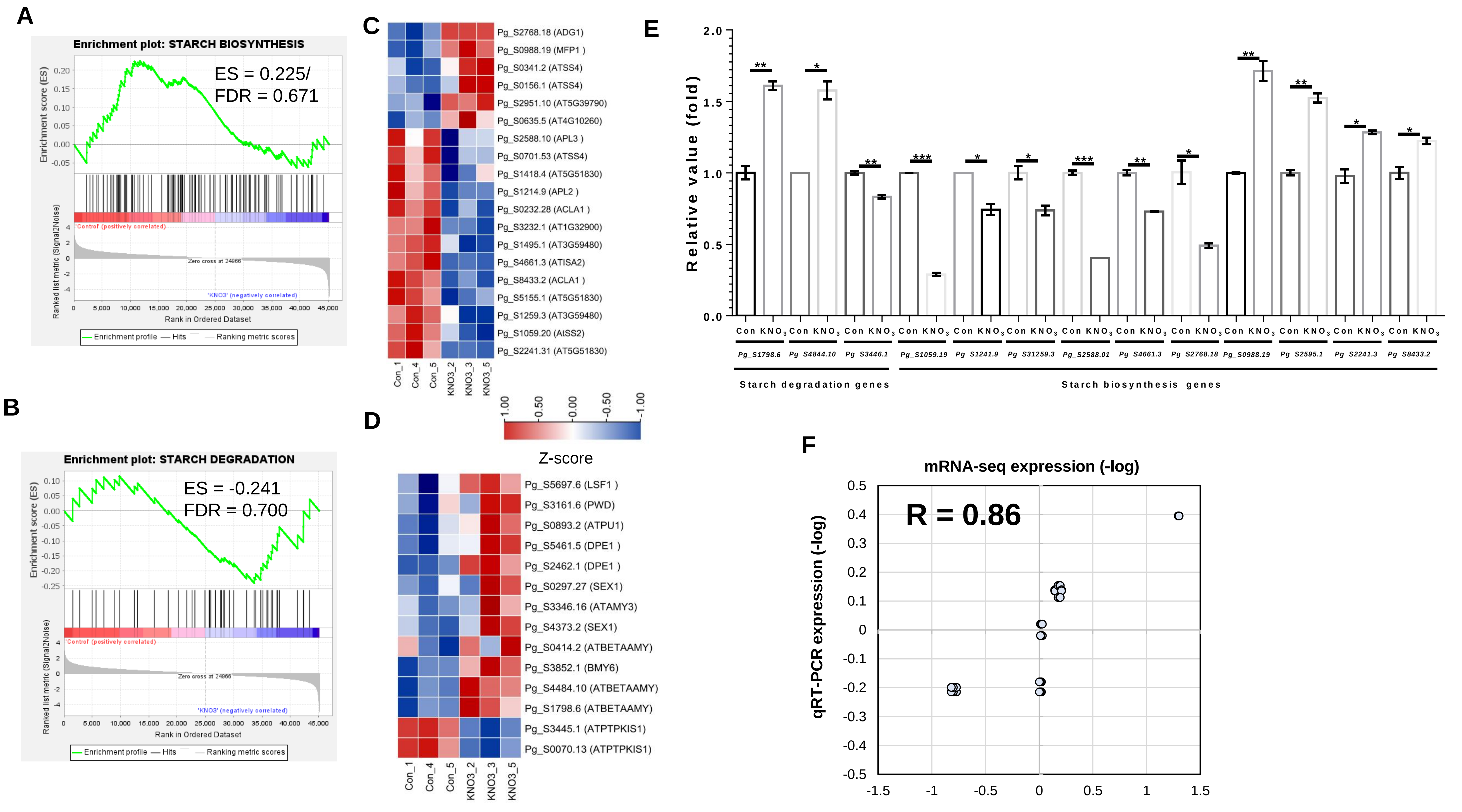
A
Enrichment plot: STARCH BIOSYNTHESIS
ES = 0.225/
FDR = 0.671
Enrichment score (ES)
Ranked list metric (Signal2Noise)
'Control' (positively correlated)
Zero cross at 24966
'KNO3' (negatively correlated)
Rank in Ordered Dataset
Enrichment profile — Hits — Ranking metric scores
B
Enrichment plot: STARCH DEGRADATION
ES = -0.241
FDR = 0.700
Enrichment score (ES)
Ranked list metric (Signal2Noise)
'Control' (positively correlated)
Zero cross at 24966
'KNO3' (negatively correlated)
Rank in Ordered Dataset
Enrichment profile — Hits — Ranking metric scores
C
Pg_S2768.18 (ADG1)
Pg_S0988.19 (MFP1)
Pg_S0341.2 (ATSS4)
Pg_S0156.1 (ATSS4)
Pg_S2951.10 (AT5G39790)
Pg_S0635.5 (AT4G10260)
Pg_S2588.10 (APL3)
Pg_S0701.53 (ATSS4)
Pg_S1418.4 (AT5G51830)
Pg_S1214.9 (APL2)
Pg_S0232.28 (ACLA1)
Pg_S3232.1 (AT1G32900)
Pg_S1495.1 (AT3G59480)
Pg_S4661.3 (ATISA2)
Pg_S8433.2 (ACLA1)
Pg_S5155.1 (AT5G51830)
Pg_S1259.3 (AT3G59480)
Pg_S1059.20 (AtSS2)
Pg_S2241.31 (AT5G51830)
Con_1 Con_4 Con_5 KNO3_2 KNO3_3 KNO3_5
1.00 0.50 0.00 -0.50 -1.00
Z-score
D
Pg_S5697.6 (LSF1)
Pg_S3161.6 (PWD)
Pg_S0893.2 (ATPU1)
Pg_S5461.5 (DPE1)
Pg_S2462.1 (DPE1)
Pg_S0297.27 (SEX1)
Pg_S3346.16 (ATAMY3)
Pg_S4373.2 (SEX1)
Pg_S0414.2 (ATBETAAMY)
Pg_S3852.1 (BMY6)
Pg_S4484.10 (ATBETAAMY)
Pg_S1798.6 (ATBETAAMY)
Pg_S3445.1 (ATPTPKIS1)
Pg_S0070.13 (ATPTPKIS1)
Con_1 Con_4 Con_5 KNO3_2 KNO3_3 KNO3_5
E
Relative value (fold)
Con KNO3
Pg_S1798.6 Pg_S4844.10 Pg_S3446.1 Pg_S1059.19 Pg_S1241.9 Pg_S31259.3 Pg_S2588.01 Pg_S4661.3 Pg_S2768.18 Pg_S0988.19 Pg_S2595.1 Pg_S2241.3 Pg_S8433.2
Starch degradation genes
Starch biosynthesis genes
F
mRNA-seq expression (-log)
R = 0.86
qRT-PCR expression (-log)

Supplementary Figure S4 . Expression of starch biosynthesis and degradation related genes are correlated with nitrate-mediated inhibition of starch granule development. (A) Enrichment plot for starch biosynthetic genes (GO:0019252, Starch biosynthetic process). (B) Enrichment plot for starch degradation related genes (GO:0005983, Starch catabolic process). (A,B) ES indicates enrichment score from GSEA analysis. (C) Expression heatmap of DEGs in starch biosynthetic genes (D) Expression heatmap of starch degradation related genes. (E) Starch degradation and biosynthesis relate genes were analyzed by qRT-PCR. *PgACT* was used as an internal control. Error bars indicate SEM (*P < 0.05, **P < 0.01 ***P < 0.001, Student's t-test).). (F) Linear regression analysis between qRT-PCR expression and mRNA-seq data. Relative expression levels of each gene were determined by $2^{-\Delta\Delta CT}$ method, as indicated in the x axis, while the TMM-normalized TPM of target genes divided by that of *PgACT* is indicated in the y axis.
